# Age-associated elevation in TLR5 leads to increased inflammatory responses in the elderly

**DOI:** 10.1111/j.1474-9726.2011.00759.x

**Published:** 2011-11-28

**Authors:** Feng Qian, Xiaomei Wang, Lin Zhang, Shu Chen, Marta Piecychna, Heather Allore, Linda Bockenstedt, Stephen Malawista, Richard Bucala, Albert C Shaw, Erol Fikrig, Ruth R Montgomery

**Affiliations:** 1 Section of Rheumatology300 Cedar Street, New Haven, CT 06520, USA; 2 Program on Aging300 Cedar Street, New Haven, CT 06520, USA; 3 Section of Infectious Diseases, Department of Internal Medicine, Yale University School of Medicine300 Cedar Street, New Haven, CT 06520, USA; 4 The Howard Hughes Medical Institute300 Cedar Street, New Haven, CT 06520, USA

**Keywords:** aging, toll-like receptors, monocytes, immunosenescence, IL-8, flagellin

## Abstract

Aging is accompanied by a progressive decline in immune function. Studies have shown age-related decreases in the expression and signaling efficiency of Toll-like receptors (TLRs) in monocytes and dendritic cells and dysregulation of macrophage TLR3. Using a multivariable mixed effect model, we report a highly significant increase in TLR5-induced production of IL-8 from monocytes of older individuals (*P* < 0.0001). Elevated IL-8 is accompanied by increased expression of TLR5, both protein and mRNA, and by increased levels of TLR5-mediated phosphorylation of MAPK p38 and ERK. We noted incomplete activation of NF-κB in response to TLR5 signaling in monocytes of elderly donors, as reflected by the absence of an associated increase in the production of TNF-α. Elevated TLR5 may provide a critical mechanism to enhance immune responsiveness in older individuals.

## Introduction

Aging is associated with a progressive decline in immune function (immunosenescence), resulting in an increased susceptibility to infection and decreased response to vaccines (Pawelec, 1999). The adaptive immune system is affected by aging, with a well-documented dysregulation in humoral as well as cell-mediated immune responses (McGlauchlen & Vogel, 2003; Linton & Dorshkind, 2004). Recent studies have begun to document the impact of aging on the innate immune system, but age-related deficits, especially in human subjects, remain incompletely defined (Rosenstiel *et al.*, 2008; Shaw *et al.*, 2010).

Toll-like receptors (TLRs) are key components of the innate immune system that detect pathogen components and trigger antimicrobial host defense responses (Kawai & Akira, 2007). Recognition via TLRs initiates signal transduction pathways that control innate immune responses and facilitate the development of antigen-specific adaptive immunity (Takeda & Akira, 2005). Studies have shown reduced surface expression of TLRs 1 and 4 in monocytes of elderly human donors that correlates with an age-associated reduction in TLR1/2 function, and a reduction in costimulatory responses that was associated with decreased antibody responses to influenza vaccination in older donors (van Duin *et al.*, 2007a,b). Reduced expression of TLRs and TLR signaling in primary dendritic cells (DCs) from elderly subjects was highly correlated with reduced influenza vaccine response (Panda *et al.*, 2010). *In vitro* infection with West Nile virus showed a reduced production of type I interferon from DCs of older donors (Qian *et al.*, 2011), and a dysregulation of TLR3 in macrophages of elderly donors (Kong *et al.*, 2008). Thus, reduced levels of certain TLRs and diminished TLR signaling likely contribute to impaired immune responses, and the increases in morbidity and mortality noted in elderly populations (Shaw *et al.*, 2011).

In an effort to improve vaccination efficiency, which is a critical need in elderly populations, several influenza vaccine formulations currently in clinical trials employ bacterial flagellin, which is an agonist for TLR5 (Bates *et al.*, 2008; Huleatt *et al.*, 2008; Skountzou *et al.*, 2009; Taylor *et al.*, 2011). In the current study, we have investigated the effects of aging on expression and function of TLR5.

## Results

### Elevated TLR5-mediated IL-8 production by monocytes from older donors

Monocytes develop in the bone marrow and circulate in the blood stream until they are recruited to extravascular compartments (Leon & Ardavin, 2008). We have previously used flow cytometry to assess monocytes in peripheral blood mononuclear cells (PBMCs) from older and younger donors and have documented age-related reduction in the expression and function of TLRs 1 and 4 in monocytes (van Duin *et al.*, 2007b). To further investigate the effects of aging on TLR function, we have assessed age-related effects on signaling efficiency of monocytes stimulated by TLR ligands. As has been shown previously (Seidler *et al.*, 2010), we detected comparable numbers of monocytes in PBMCs from donors of both age groups (younger age group 17.02 ± 0.64%, *n* = 145 vs. older age group 18.36 ± 0.59%, *n* = 197; *P* = 0.13).

**Figure 1:**
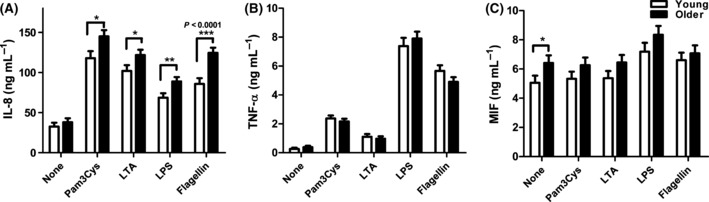
Age-associated alterations in cytokine and chemokine production in monocytes. Monocytes from older and young adults were stimulated with indicated toll-like receptor ligands for 20 h. IL-8 (A, older *n* = 203, young *n* = 141), TNF-α (B, older *n* = 203, young *n* = 141), and MIF (C, older *n* = 139, young *n* = 105) were quantified from culture supernatants by ELISA. Data shown are the means ± SEM; asterisks indicate statistical significance between young and older cohort (*P < 0.05, **P < 0.01 and ***P < 0.001).

To investigate the effects of aging on human TLR function, we stimulated purified, adherent monocytes overnight with TLR ligands as follows: Pam3CSK4 (TLR1/2), LTA (TLR2/6), LPS (TLR4), and flagellin (TLR5). For these analyses, we focused on IL-8 and TNF-α production, cytokines classically associated with TLR engagement, and macrophage migration inhibitory factor, MIF, a cytokine that amplifies the pro-inflammatory milieu (Metz & Bucala, 1997). At baseline, levels of IL-8 and TNF-α were comparable between the two age groups, while the MIF levels were higher in monocytes from older donors (Fig. 1). After 20 h of stimulation with TLR ligands, monocytes from both age groups showed induction of IL-8 and TNF-α. Using a multivariable mixed effects statistical model, we observed statistically significant, elevated levels of IL-8 production in cells from older (>60 years), compared to young adults aged 21–39 years, following the engagement of TLR1/2, TLR2/6, TLR4, and TLR5; the highest magnitude change was observed following flagellin stimulation, which was increased by 45.1% (Fig. 1A; *P* < 0.0001). The least squared mean difference was highly statistically significant after adjustment for covariates. None of the other differences noted between older and young adults, e.g., as observed for TNF-α and MIF responses after stimulation with TLR ligands, reached significance between age groups.

### Age-associated alterations in TLR5 expression in human monocytes

**Figure 2:**
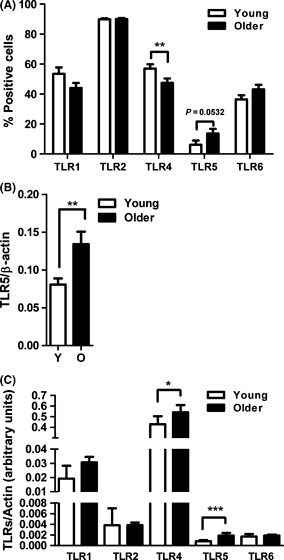
Age-associated alterations in toll-like receptor (TLR) expression in human monocytes. (A) Data shown are percent-positive monocytes for TLR1 (older *n* = 119, young *n* = 78), TLR2 (older *n* = 116, young *n* = 78), TLR4 (older *n* = 118, young *n* = 79), TLR5 (older *n* = 38, young *n* = 47), and TLR6 (older *n* = 90, young *n* = 69) surface expression. Values indicate the means ± SEM in young and older adults; asterisks indicate statistical significance between young and older cohort (**P < 0.01). (B) Densitometry of immunoblot of TLR5 in monocytes from 21 pairs of younger and older subjects. Densitometry shows the means ± SEM of the ratio of TLR5 to β-actin. Asterisks indicate statistical significance between younger and older cohort (**P < 0.01). (C) The data display mRNA expression levels of the indicated TLRs in purified monocytes from young (*n* = 23) and older adults (*n* = 26). mRNA levels were quantified by qPCR and normalized to β-actin. Data shown are the median with interquartile range; asterisks indicate statistical significance between young and older cohort (*P < 0.05 and ***P < 0.001).

To determine whether a concomitant alteration of TLR expression might account for the age-associated increase in TLR5-mediated cytokine response, we quantified surface expression of TLRs 1, 2, 4, 5, and 6 from monocytes of younger and older individuals by flow cytometry (Fig. 2A). We detected decreased expression of TLR1 and TLR4 (*P* < 0.01) and unchanged levels of TLR2 in monocytes from older individuals, consistent with our previous results (van Duin *et al.*, 2007b) and no significant age-related changes in expression of TLR6. In contrast, surface expression of TLR5 was higher in monocytes from older individuals than from younger individuals (Fig. 2A, *P* = 0.0532). Additionally, western blot analysis on monocyte cell lysates revealed significantly higher total cell TLR5 protein level from older donors (Fig. 2B, *P* = 0.0053).

We have previously shown that both transcriptional and post-translational effects contribute to the decreases noted in TLR function in DCs in aging (Panda *et al.*, 2010). Thus, to assess a role of transcriptional mechanisms in the observed age-associated differences in TLR expression in monocytes, we assessed TLR mRNA expression levels using qPCR of highly purified monocyte populations from the younger and older groups. When comparing mRNA expression, we detected significantly increased expression of TLR4 and TLR5 by monocytes from older individuals whereas levels of TLR1, TLR2, and TLR6 were comparable to those from younger individuals (Fig. 2C). Of note, only TLR5 had elevated mRNA and surface expression on monocytes from older donors as compared with young adults, which may contribute to the observed age-associated alterations in TLR5 function.

### Age-related increases in TLR5-mediated MAPK signaling pathways

**Figure 3:**
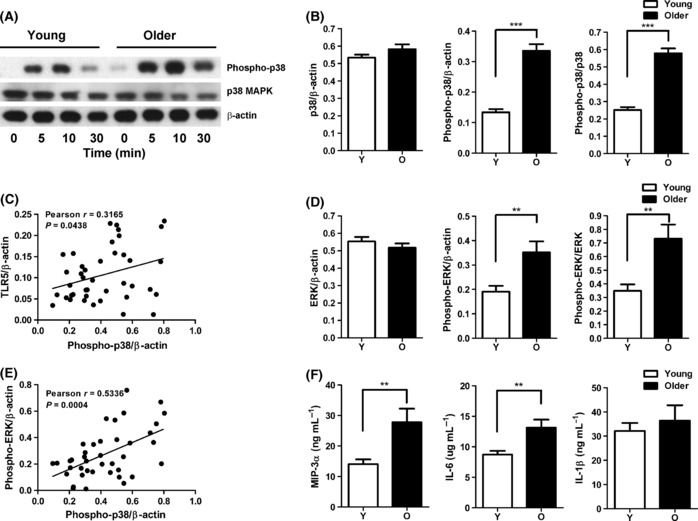
Flagellin-stimulated monocytes from the elderly show increased phosphorylation of p38 and ERK MAPK. (A) immunoblot of p38 phosphorylation in a representative result of young and old individuals after stimulation with the toll-like receptor (TLR)5 ligand flagellin in monocytes. Phosphorylation levels at 5, 10, and 30 min are compared with unstimulated control samples. (B) Densitometry of immunoblot of p38 and phospho-p38 in monocytes from 21 pairs of younger and older subjects after stimulation with flagellin for 10 min. Densitometry shows the means ± SEM of the ratio of total p38 and phospho-p38 to β-actin, and of phospho-p38 to total p38. Asterisks indicate statistical significance between younger and older cohort (***P < 0.001). (C) Correlation of TLR5 expression with p38 phosphorylation after stimulation with flagellin (*n* = 42, Pearson *r* = 0.3165, *P* = 0.0438). (D) Densitometry of immunoblot of ERK in monocytes from 21 pairs of younger and older subjects after stimulation with flagellin for 10 min. Densitometry shows the means ± SEM of the ratio of total ERK and phospho-ERK to β-actin, and of phospho-ERK to total ERK. Asterisks indicate statistical significance between younger and older cohort (**P < 0.01). (E) Correlation of ERK phosphorylation with p38 phosphorylation after stimulation with flagellin (*n* = 42, Pearson *r* = 0.5336, *P* = 0.0004). (F) Monocytes from older (*n* = 20) and young (*n* = 20) adults were stimulated with TLR5 ligand flagellin for 20 h. IL-1β, IL-6, and MIP-3α were measured from culture supernatants by ELISA. Data shown are the means ± SEM; asterisks indicate statistical significance between young and older cohort (**P < 0.01).

To define the mechanism underlying the age-related increase in the production of IL-8 in response to TLR5 stimulation, we assessed the activation of key signaling pathways involved in the generation of cytokine responses. TLR5 stimulation results in phosphorylation of p38 and ERK MAPKs (mitogen-activated protein kinases) to active forms (Zeng *et al.*, 2006), thus we assessed the activation level of the MAPK p38 and ERK in monocytes of both age groups. Monocytes from both age groups (adherent for 2 h) stimulated with flagellin showed the activation of p38 and increased levels of phospho-p38 noted as early as 5 min and throughout the 30-min time course (Fig. 3A). However, the increase in p38 phosphorylation was significantly higher in monocytes from older donors compared with younger donors and was sustained longer compared with young donors (Fig. 3A,B). The ratio of phospho-p38 was elevated relative both to β-actin, representing total cell protein, as well as to total p38 (densitometry at 10 min of stimulation, Fig. 3B). In addition, a significant correlation was observed between increased TLR5 expression and p38 phosphorylation (Fig. 3C, Pearson *r* = 0.3165, *P* = 0.0438). A similar increase in the activation of the signaling kinase ERK was noted when monocytes of older donors were stimulated through TLR5 (Fig. 3D), and the increase in the phosphorylation of p38 and ERK was related as shown by a correlation analysis (Fig. 3E, Pearson *r* = 0.5336, *P* = 0.0004). We also assessed the age-related difference in phospho-p38 in the monocytes from older and younger donors after LPS stimulation. No statistically significant age-related differences were noted (densitometry of phospho-p38; younger 0.3520 ± 0.0268 vs. older 0.3476 ± 0.0207; 12 subjects/group; *P* = 0.8983), indicating the elevated p38 phosphorylation in monocytes from older donors was specific for flagellin stimulation.

Increased activation of p38 and ERK would be expected to lead to an increase in the production of cytokines and chemokines, including increased production of IL-8 noted in older donors. We extended these findings in a subset of donors and quantified the production of MIP-3α, IL-6, and IL-1β, additional target genes expected to be induced as a result of TLR5 engagement and phosphorylation of p38 and ERK (Rhee *et al.*, 2004). We observed significant increases in the levels of MIP-3α and IL-6, as detected by ELISA from monocytes in a subset of older donors (*n* = 20/group) after stimulation of TLR5 (Fig. 3F) and these differences correlated with increased levels of phosphorylated p38 (IL-8 Pearson *r* = 0.5257, *P* = 0.0005; MIP-3α Pearson *r* = 0.5640, *P* = 0.0002; IL-6 Pearson *r* = 0.5756, *P* = 0.0001). However, no significant difference was observed in the monocyte production of IL-1β and TNF-α between the young and older adults, suggesting that there are other factors regulating TLR5 signaling.

### Monocytes from older donors do not fully activate NF-κB pathway

**Figure 4:**
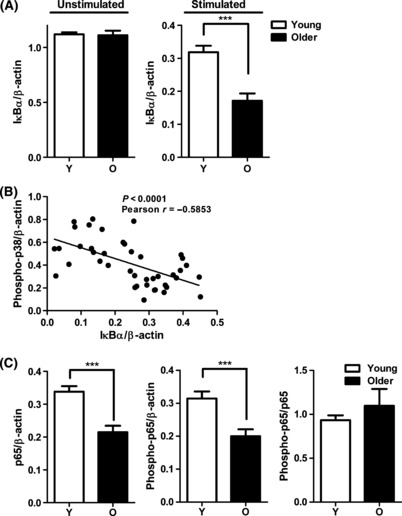
Monocytes from older donors do not fully activate NF-κB pathway (A) A significant increase in IκBα degradation in monocytes from older donors. Densitometry of immunoblot of IκBα in monocytes from younger and older subjects before stimulation (*n* = 12/group) and after stimulation with Flagellin for 10 min (*n* = 21/group). Densitometry shows the means ± SEM of the ratio of total IκBα to β-actin. Asterisks indicate statistical significance between younger and older cohort (***P < 0.001). (B) Degradation of IκBα correlated with p38 phosphorylation in monocytes after stimulation with flagellin (*n* = 42, Pearson *r* = −0.5853, *P* < 0.0001). (C) The decrease in total NF-κB p65 and phospho-p65 expression in monocytes from older individuals. Densitometry of immunoblot of total p65 and phospho-p65 in monocytes from 21 pairs of younger and older subjects after stimulation with flagellin for 10 min. Densitometry shows the means ± SEM of the ratio of total p65 and phospho-p65 to β-actin. Asterisks indicate statistical significance between younger and older cohort (***P < 0.001).

To identify other regulators that may contribute to elevated TLR5-mediated responses in monocytes from elderly donors, we examined the NF-κB pathway, a pivotal regulator and downstream target of innate immune receptors and cytokines. IκBα is a multifunctional inhibitor of NF-κB, blocking nuclear translocation, DNA binding, and phosphorylation (Jacobs & Harrison, 1998). When levels of IκBα were quantified by immunoblot, no significant difference was observed between age groups before stimulation. However, significantly more degradation of IκBα was noted in monocytes from older donors after flagellin stimulation (Fig. 4A). The increased degradation of IκBα correlated with the increased phosphorylation of p38 in monocytes from older donors (Fig. 4B), indicating an elevated signaling activation after flagellin stimulation of monocytes from older donors.

Degradation of IκBα relieves the inhibition of NF-κB, leading to phosphorylation of the active component NF-κB (p65). Thus, the lower levels of IκBα in monocytes from older donors would be expected to lead to higher levels of phosphorylation of NF-κB p65. Surprisingly, quantitative immunoblot analysis of NF-κB p65 showed lower levels of both total and phospho-NF-κB p65 from monocytes of older donors (Fig. 4C). This finding suggests the presence of an additional age-related dysregulation that may block the full activation of NF-κB pathway in older donors.

**Figure 5:**
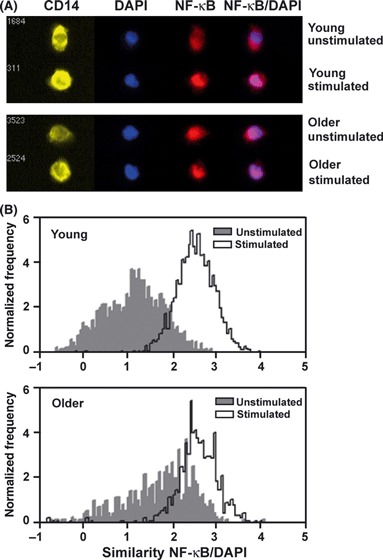
Nuclear translocation of NF-κB p65 in monocytes by ImageStream. Peripheral blood mononuclear cells from young (*n* = 8) and older (*n* = 10) subjects were stimulated for the indicated times with flagellin (2.5 μg mL^−1^), and images were collected on the ImageStream cytometer. Data were analyzed to determine the percentage of CD14+ monocytes with ‘similarity’ defined as NF-κB p65 translocated and colocalizing with nuclear stain (DAPI). (A) Representative image from cells before (untreated) and following flagellin stimulation. (B) Histogram examples of ‘similarity’ score from unstimulated (gray) monocytes and flagellin stimulated (white) from younger and older subjects.

After the complete degradation of IκBα, NF-κB (p65) is translocated to the nucleus to modulate the expression of target genes. To quantify nuclear translocation of NF-κB in activated monocytes, we used the ImageStream technology that combines high-resolution digital imaging with quantitative flow cytometry technology. We used the IDEAs software ‘similarity’ feature to quantify the overlap (or ‘similarity’) of staining of NF-κB (p65) with nuclear staining (DAPI) so that a high correlation of NF-κB/DAPI localization is reflected in a high similarity score. When we quantified the age-related differences in nuclear localization of NF-κB (p65) in CD14+ monocytes, we observed a significantly higher percentage of unstimulated cells from older donors with NF-κB (p65) translocated into the nucleus in cells, i.e., a higher similarity score at baseline (Fig. 5; older 25.26 ± 3.99%, *n* = 10 vs. younger 14.56 ± 1.45%, *n* = 8; *P* = 0.0361). However, after stimulation with flagellin, the percentage of cells with NF-κB (p65) translocated to the nucleus was comparable in monocytes between age groups (older 82.62 ± 2.85%, *n* = 10 vs. younger 78.19 ± 4.21%, *n* = 8; *P* = 0.3819). Because the baseline levels were elevated in cells from older donors, the effective fold change in NF-κB translocation was lower in older donors (older 3.75 ± 0.42, *n* = 10 vs. younger 5.73 ± 1.59%, *n* = 8; *P* = 0.0134). These results suggest a previously unrecognized dysregulation of the NF-κB pathway in monocytes from older donors.

## Discussion

We quantified the age-related production by monocytes of the cytokines IL-8, TNF-α, and MIF following stimulation with a wide range of TLR ligands. While unstimulated levels of IL-8 and TNF-α were equivalent between age groups, macrophage migration inhibitory factor (MIF), a cytokine with protean influences on inflammation, had higher basal levels in monocytes from older donors. In addition, we observed a higher representation at baseline of the signaling mediator NF-κB (p65) translocated to the nucleus of monocytes from older donors. These results are consistent with the increased proinflammatory milieu associated with elevated levels of cytokines in older individuals, so called inflammaging, or chronic, low-grade inflammation which may contribute to age-associated frailty, morbidity, and mortality (Franceschi *et al.*, 2007).

While we observed stimulation-specific statistically significant increases in the production of IL-8 by adherent monocytes from older individuals for all TLR ligands tested, the magnitude of increase in IL-8 production was highest following treatment with the TLR5 ligand, flagellin. This is distinct from our study of mixed PBMCs assayed by FACS in suspension (van Duin *et al.*, 2007b), in which cells from older donors showed lower responses to all TLR ligands tested, and is consistent with enhanced responsiveness noted with progressive differentiation (Mizel & Bates, 2010). The elevated production of IL-8 after flagellin stimulation was accompanied by higher levels of TLR5 as quantified by surface protein expression and mRNA levels in monocytes from older adults. In our previous study, we did not observe any difference in TLR5 mRNA level in mDC (myeloid DCs) between younger and older groups (Panda *et al.*, 2010), suggesting that the higher expression of TLR5 from older donors is cell type specific. While the mechanisms of the age-related effect on TLR5 expression are currently unknown, future studies will examine the contributions of transcriptional and translational regulatory events, alterations in TLR5 localization, and possible roles of known and as yet unidentified TLR chaperone or accessory molecules, which are increasingly understood to be critical for the expression of TLRs, i.e. PRAT4A, UNC-93B, and gp 96 (Conley, 2007; Takahashi *et al.*, 2007; Akashi-Takamura & Miyake, 2008).

Stimulation of TLR5 by flagellin is mediated through the signaling mediators MAPK p38 and ERK, which also were elevated in the monocytes of older compared with younger donors. The increased production of IL-8, IL-6, and MIP-3α noted here is consistent with higher expression of TLR5 in the elderly and strongly correlated with increased levels of p38 MAPK activation, suggesting that TLR5 activation is a critical mechanism contributing to the increase in flagellin-stimulated IL-8 production in monocytes from older vs. younger donors. This is consistent with previous reports documenting a role of p38 in post-transcriptional regulation of IL-8 mRNA (Hoffmann *et al.*, 2002; Yu *et al.*, 2003). No correlation was noted between NF-κB (p65) phosphorylation and increased production of IL-8 in older monocytes, suggesting that TLR5-mediated p38 activation and subsequent IL-8 expression occur independently of NF-κB activation. Although we observed lower levels of IκBα, the endogenous inhibitor of NF-κB, in monocytes from older donors, the degradation of IκBα did not support full activation of NF-κB signaling in elderly. A recent study in airway mucosa identifies a role for dual oxidase 2 in flagellin-medicated activation of NF-κB (Joo *et al.*, 2011). Future studies will investigate the further dysregulation of NF-κB pathway in older donors.

The TLR5 ligand flagellin is a potent activator of a broad range of cell types involved in innate and adaptive immunity (Feuillet *et al.*, 2006). Flagellin administration can induce prominent local and systemic immune/inflammatory responses *in vivo* that may provide a clinically useful approach for treating antibiotic-associated intestinal infections (Kinnebrew *et al.*, 2010) and enhancing cancer radiotherapy (Burdelya *et al.*, 2008). The incorporation of flagellin into vaccines has been shown to enhance the immune response in influenza vaccines and cancer immunotherapeutic studies (Skountzou *et al.*, 2009; Song *et al.*, 2009; Faham & Altin, 2010).

The elevated levels of TLR5 in elderly donors suggest the possibility of enhancing immune responses to vaccination through targeting TLR5. Indeed, in both murine models (Bates *et al.*, 2008; Leng *et al.*, 2011) and recent clinical trials (Taylor *et al.*, 2011), including flagellin in the vaccine formulation enhanced vaccination efficiency in elderly subjects. Elderly donors immunized with a flagellin-conjugated influenza virus reached an equivalent titer with a smaller dose than either standard or high-dose flu vaccination reported previously for subjects >65 years (Falsey *et al.*, 2009), suggesting enhanced efficiency through targeting TLR5 (Taylor *et al.*, 2011). Our current finding on age-associated elevation in TLR5-mediated immune responses offers novel opportunities for flagellin-related therapeutic uses and vaccines – a potentially powerful strategy to harness the innate immune system to address the increased susceptibility to infections and decreased response to vaccines in the elderly.

## Experimental procedures

### Human subjects

Heparinized blood from healthy volunteers was obtained after written informed consent under the guidelines of the Human Investigations Committee of Yale University. Donors had no acute illness and took no antibiotics or nonsteroidal anti-inflammatory drugs within 1 month of enrollment. Young donors (*n* = 168) were aged 26.2 years (range 21–39), 59.5% female and 74.4% White, 7.7% Black, and 16.1% Asian. Older donors (*n* = 221) were 73.4 years (range 60–93), 54.8% female and 91.9% White, 5.9% Black, 1.4% Asian. Self-reported information included demographic data, height, weight, medications, and comorbid conditions; immunocompromised individuals as defined previously were excluded (Panda *et al.*, 2010).

### Monocyte isolation and stimulation

Human PBMCs were isolated using Ficoll-Hypaque (GE Healthcare, Piscataway, NJ, USA) (Kong *et al.*, 2008). Cells were plated at 0.5 × 10^6^ PBMCs/well into 48-well plates or 5 × 10^6^ PBMCs/well into 6-well plates (BD Falcon, San Jose, CA, USA). After 2 h, nonadherent cells were removed by washing and adherent monocytes were stimulated for 20 h with TLR ligands Pam3CSK4 (5 μg mL^−1^; TLR1/2), LTA (1 μg mL^−1^; TLR2/6), LPS (0.5 μg mL^−1^; TLR4), and flagellin from *Salmonella typhimurium* (2.5 μg mL^−1^; TLR5) (InvivoGen, San Diego, CA, USA).

### Cytokine ELISA measurements

Supernatants from monocytes were stored frozen, and cytokines were quantified by batch analysis enzyme-linked immunosorbent assays (ELISA) (OptEIA ELISA kit; BD Biosciences, San Jose, CA, USA). Human MIF was quantified by specific capture ELISA, employing native sequence MIF as standard (McDevitt *et al.*, 2006).

### Flow cytometry

Peripheral blood mononuclear cells (1.0 × 10^6^) were labeled on the day of isolation for 30 min at 4 °C as follows: TLR1-PE, TLR2-FITC, TLR4-PE, TLR6-biotin, CD4-PE-Cy5 (eBioscience, San Diego, CA, USA), and goat anti-human-TLR5 (Alexis Biochemicals, San Diego, CA, USA). Streptavidin-PE (BD Biosciences) for TLR6-biotin and FITC donkey anti-goat IgG (Jackson ImmunoResearch, West Grove, PA, USA) for TLR5. Cells were washed in PBS/2% FBS, and 40 000 events/tube were acquired using a FACSCalibur instrument (BD Biosciences) and analyzed using FlowJo software (Tree Star, Ashland, OR, USA). Monocytes were identified by forward- and side-scatter combined with surface staining (CD4 dim) (Alexopoulou *et al.*, 2002).

### Quantitative PCR (qPCR) analysis

CD14+ monocytes were isolated from PBMC using a MACS system (Miltenyi Biotech, Auburn, CA, USA). Total RNA was harvested by RNeasy mini kit (Qiagen, Valencia, CA, USA), and cDNA was synthesized using AffinityScript Multi Temperature cDNA Synthesis Kit (Stratagene, Cedar Creek, TX, USA). Primers and probes for qPCRs were from Applied Biosystems (Foster City, CA, USA): TLR1 (Hs00413978_m1), TLR2 (Hs01014511_m1), TLR4 (Hs00152939_m1), TLR5 (Hs01920773_s1), and TLR6 (Hs00271977_s1) or synthesized as described previously (Kong *et al.*, 2008). Amplification in duplicate was on batched samples in an iCycler (Bio-Rad, Hercules, CA, USA); values were normalized to β-actin (Kong *et al.*, 2008).

### Immunoblot analysis

Peripheral blood mononuclear cells were plated (5 × 10^6^/35 mm well) for 2 h, washed, and adherence-purified monocytes stimulated with flagellin (2.5 μg mL^−1^) for 0–30 min were harvested using CelLytic M Cell Lysis buffer (Sigma, St. Louis, MO, USA) containing protease inhibitor cocktail (Kong *et al.*, 2008). Immunoblots were probed with anti-phospho-p38 MAPK, anti-phospho-ERK, anti-phospho-p65, anti-IκBa, and anti-β-actin, developed using Amersham ECL Reagents (GE Healthcare), and scanned using Image J software.

### ImageStream quantitation of nuclear translocation

Peripheral blood mononuclear cells (1.5 × 10^6^/tube) were stimulated with flagellin (2.5 μg mL^−1^) at 37 °C. Cells were fixed in BD Phosflow Fix buffer I (BD Biosciences), resuspended in 90% FBS containing 10% DMSO, and stored at −80 °C. For analysis, batches of cells were labeled with CD14-PE (eBioscience), permeabilized in BD Perm/Wash buffer (BD), and labeled with rabbit anti-NF-κB (p65) antibody (SantaCruz Biotechnology, Santa Cruz, CA, USA) for 20 min at RT and detected using goat anti-rabbit IgG-Alexa647 (Invitrogen, Carlsbad, CA, USA) and incubating for 20 min at RT. Nuclei were counterstained with DAPI (0.2 μg mL^−1^, Invitrogen) prior to imaging by ImageStream and analyzed using ideas software (Amnis, Seattle, WA, USA).

### Statistical analysis

Demographic characteristics of subjects were collected at enrollment (van Duin *et al.*, 2007b; Panda *et al.*, 2010). Cytokine responses used mixed effects models adjusting for year of sampling, sex and race, ligand-and-ligand by age group interaction. To address human heterogeneity, we used an unstructured covariance structure for each person to have a unique correlation among TLRs. For surface expression data, we used multivariable general linear models to estimate the effect of age group on the percent-positive cells expressing specific TLRs; least squared means are presented to improve clinical interpretation. Statistical tests were 2-tailed, and *P* < 0.05 considered significance. TLR, cytokine, and vaccine response analyses used sas version 9.2 (SAS Institute, Cary, NC, USA). Difference for qPCR data was determined using Mann–Whitney test with Bonferroni correction or unpaired t test using Graphpad Prism (GraphPad Software, Inc., La Jolla, CA, USA).
